# Metric System

**Published:** 1878-08

**Authors:** 


### METRIC SYSTEM.

It is now certain that the metric system of weights and measures will be finally adopted in this country. Of its greater simplicity and its superiority, compared with our present system, there is no doubt. The difficulty is in computing in the new system relative amounts with those we have become familiar in the old. For example, we have become accustomed to give twenty grains of certain solids and twenty drops of a liquid for the doses, and when they have to be expressed in grams we are at a loss without making a calculation. This can be overcome only by practicing the new system.

The international metric system not only simplifies by using decimals, but estimates liquids as well as solids by weight when used in small quantities.

METRICAL WEIGHTS AND THEIR EQUIVALENTS IN TROY WEIGHTS AND MEASURES.

| | | | | |
|---|---|---|---|---|
| 10 Milligrams (mg) | 1 Centigram | (cg.)= 0.15 grains= | 0.15 minims. |
| 10 Centigrams, | 1 Decigram | (dg.)= 1.54 grains= | 1.54 minims. |
| 10 Decigrams | 1 GRAM, | (gm.)=15.43 grains= | 15.43 minims. |
| 10 GRAMS | 1 Dekagram, | (Dg.)= 2.57 drms. = | 2.57 fl drms. |
| 10 Dekagrams | 1 Hectogram, | (Hg.)= 3.21 oz. = | 3.21 fl. oz. |
| 10 Hectograms | 1 Kilogram, | (Kg.)=32.14 oz. = | 32.14 fl. or 2 O. |

A box about one-third of an inch, or one centiliter square, will hold nearly 15½ minims of water, which weighs nearly 15½ Troy grains. The gram weight will balance this amount, and is therefore the equivalent of 15½ minims. It seems, therefore, that a minim of water weighs one grain, and as 60 minims make a fluidrachm, and 60 grains a Troy drachm, the gram will serve for either, and hence the above table. Four grams will make about one teaspoonful, and sixteen grams a tablespoonful. The above table is sufficient for compounding prescriptions of fluids and solids; and the annexed equivalents in the old familiar weights and measures will enable physicians and apothecaries to estimate the gram and its divisions, until they also become familiar.

